# Performances of whole-brain dynamic and static functional connectivity fingerprinting in machine learning-based classification of major depressive disorder

**DOI:** 10.3389/fpsyt.2022.973921

**Published:** 2022-07-26

**Authors:** Heng Niu, Weirong Li, Guiquan Wang, Qiong Hu, Rui Hao, Tianliang Li, Fan Zhang, Tao Cheng

**Affiliations:** ^1^Department of MRI, Shanxi Cardiovascular Hospital, Taiyuan, China; ^2^Department of Neurology, Shanxi Cardiovascular Hospital, Taiyuan, China; ^3^Department of Ultrasound, Shanxi Cardiovascular Hospital, Taiyuan, China; ^4^Department of Medical Imaging, Shanxi Traditional Chinese Medical Hospital, Taiyuan, China

**Keywords:** major depressive disorder, resting-state functional magnetic resonance imaging, dynamic functional connectivity, static functional connectivity, machine learning

## Abstract

**Background:**

Alterations in static and dynamic functional connectivity during resting state have been widely reported in major depressive disorder (MDD). The objective of this study was to compare the performances of whole-brain dynamic and static functional connectivity combined with machine learning approach in differentiating MDD patients from healthy controls at the individual subject level. Given the dynamic nature of brain activity, we hypothesized that dynamic connectivity would outperform static connectivity in the classification.

**Methods:**

Seventy-one MDD patients and seventy-one well-matched healthy controls underwent resting-state functional magnetic resonance imaging scans. Whole-brain dynamic and static functional connectivity patterns were calculated and utilized as classification features. Linear kernel support vector machine was employed to design the classifier and a leave-one-out cross-validation strategy was used to assess classifier performance.

**Results:**

Experimental results of dynamic functional connectivity-based classification showed that MDD patients could be discriminated from healthy controls with an excellent accuracy of 100% irrespective of whether or not global signal regression (GSR) was performed (permutation test with *P* < 0.0002). Brain regions with the most discriminating dynamic connectivity were mainly and reliably located within the default mode network, cerebellum, and subcortical network. In contrast, the static functional connectivity-based classifiers exhibited unstable classification performances, i.e., a low accuracy of 38.0% without GSR (*P* = 0.9926) while a high accuracy of 96.5% with GSR (*P* < 0.0002); moreover, there was a considerable variability in the distribution of brain regions with static connectivity most informative for classification.

**Conclusion:**

These findings suggest the superiority of dynamic functional connectivity in machine learning-based classification of depression, which may be helpful for a better understanding of the neural basis of MDD as well as for the development of effective computer-aided diagnosis tools in clinical settings.

## Introduction

Complex psychiatric symptoms have recently been mapped to brain networks, also termed connectome, that facilitate the effective segregation and integration of information processing (1). Connectome localizations of different focal lesions that cause the same symptom may provide new insight into treatment targets for psychiatric symptoms. Functional magnetic resonance imaging (fMRI) has emerged as a non-invasive imaging technique which allows researchers to measure functional connectivity, i.e., the temporal coherence of the blood-oxygen-level-dependent (BOLD) signal between different brain regions (2, 3). Human fMRI studies have demonstrated that the brain functional connectivity profiles constitute unique “neural fingerprints” with highly individualized patterns, which allows identification of individuals at the single-subject level (4–8). Delayed stabilization and individualization in the functional connectivity development may be associated with psychiatric disorders (9). There is a large body of evidence in support of the notion that functional connectivity alterations are associated with the symptomatology and therapeutic interventions of major depressive disorder (MDD) (10–16). MDD-related functional connectivity alterations involve aberrant connections between specific region pairs, abnormal connections within or between functional networks, and disrupted topological organization of the whole-brain connectome. The consistently affected functional networks include default mode, cognitive control, salience, affective, reward, and attention networks, as well as cerebellar and subcortical circuitries.

Conventionally, resting-state functional connectivity is implicitly assumed to be temporally static, indicating that the interaction between brain regions is fixed throughout a whole resting-state scan period. However, this assumption might underestimate the complex dynamics of functional connectivity across time (17–20), which reflects its ability to rapidly switch across multiple states and allows the brain to continuously sample different configurations of its functional repertoire (21, 20). Recently, an increasing number of publications have emerged to exploit the rich temporal information contained in dynamic functional connectivity (17–20, 22). These dynamic approaches have been widely applied to the research of MDD and have enjoyed significant success in revealing the functional connectivity deficits in this disorder (23–31), going beyond the static characterization.

MDD is a complex clinical entity and its diagnosis largely depends on behavioral symptoms and clinician judgment to date. However, heterogeneity within MDD and overlapping phenotypes across psychiatric diseases sometimes render it difficult to make a stable and well-defined clinical diagnosis. An effective and objective diagnostic tool for depression is greatly needed. Recently, neuroimaging approaches have been widely used to identify reliable neurobiological markers of depression (10, 12, 14, 32–36) and machine learning techniques [i.e., the use of advanced statistical and probabilistic methods to construct systems with an ability to automatically learn from big data (37)] have been frequently applied to diagnostic classification of mental illnesses (38), which suggest that a combination of neuroimaging and machine learning may facilitate an accurate discrimination of MDD patients and healthy subjects (39–41). Among multiple neuroimaging approaches, MRI in particular has demonstrated its capacity for non-invasively measuring brain structure, function and connectivity (42–45). Thus, there has been growing interest in machine learning-based classification of depression which utilizes features extracted from structural, functional and diffusion MRI (sMRI, fMRI, and dMRI) data (46, 47). Overall, the diagnostic accuracies of machine learning studies in MDD are from 45 to 99% for resting-state fMRI-derived functional connectivity, from 72 to 97% for task fMRI-derived brain activation, from 58.7 to 90.3% for sMRI-derived brain morphology, and from 71.9 to 91.7% for dMRI-derived white matter integrity, respectively (46). However, previous resting-state fMRI studies have mainly focused on static functional connectivity, which is strongly influenced by potential confounders [e.g., global signal regression (GSR) in fMRI data preprocessing (48)] and thus may result in the high variability in classification accuracy.

Here, we used a data-driven approach to characterize whole-brain dynamic and static functional connectivity patterns based on resting-state fMRI data from MDD patients and well-matched healthy controls. Our aim was to compare the performances of dynamic and static functional connectivity combined with machine learning in discriminating patients from controls at the individual subject level. Given the dynamic nature of brain activity, we expected that dynamic connectivity would outperform static connectivity in the classification accuracy and stability.

## Materials and methods

### Participants

Patients with MDD were enrolled consecutively from the psychiatric outpatient or inpatient department of the local hospital. Healthy controls were enrolled from the local community via advertisements. This work comprised a total of 142 right-handed individuals, including 71 patients and 71 controls who did not diverge on gender (Pearson Chi-square test, χ^2^ = 1.421, *P* = 0.233), age (two-sample *t*-test, *t* = –0.734, *P* = 0.464) and education (*t* = –1.717, *P* = 0.088). The diagnoses of depression were determined by two well-trained psychiatrists utilizing the Structured Interview for DSM-IV Axis I Disorders, Patient Edition (SCID-P). Controls were carefully screened to confirm an absence of any psychiatric disorders using the MINI-International Neuropsychiatric Interview. For all participants, exclusion criteria included (1) the presence of other psychiatric illnesses, e.g., bipolar disorder, schizophrenia, substance-induced mood disorder, substance abuse or dependence, and anxiety disorders; (2) a history of major neurological or physical illnesses; (3) a history of head injury with consciousness loss; (4) any contraindications for MRI including pregnancy. For healthy controls, additional exclusion criterion included a family history of psychiatric or neurological diseases among the first-degree relatives. Hamilton Rating Scale for Depression (HAMD) (49) and Hamilton Rating Scale for Anxiety (HAMA) (50) were adopted to estimate the severity of depressive and anxiety symptoms. MDD patients showed higher HAMD (*t* = 13.694, *P* < 0.001) and HAMA (*t* = 12.426, *P* < 0.001) scores than control subjects. All patients were receiving their antidepressants including serotonin norepinephrine reuptake inhibitors (SNRIs), selective serotonin reuptake inhibitors (SSRIs), or noradrenergic and specific serotonergic antidepressant (NaSSA). This research was approved by the local ethics committee, and written informed consent was derived from all subjects after they had been given a complete description of the research. The demographic and clinical characteristics of the sample are provided in Table 1.

**TABLE 1:** Demographic and clinical characteristics.

Characteristics	MDD (*n* = 71)	HC (*n* = 71)	Statistics	*P*-value
Gender (female/male)	33/38	26/45	χ^2^ = 1.421	0.233^a^
Age (years)	40.8 ± 11.4	42.1 ± 10.1	*t* = –0.734	0.464^b^
Education (years)	10.2 ± 3.3	11.3 ± 3.9	*t* = –1.717	0.088^b^
HAMD	27.0 ± 12.0	3.8 ± 4.2	*t* = 13.694	<0.001^b^
HAMA	18.0 ± 7.6	3.9 ± 4.1	*t* = 12.426	<0.001^b^
Illness duration (months)	66.9 ± 77.8			
Onset age (years)	35.3 ± 11.7			
Episode number	2.5 ± 1.6			
**Antidepressant medications (number of patients)**				
SSRIs	47			
SNRIs	21			
NaSSA	3			
FD (mm)	0.147 ± 0.117	0.145 ± 0.086	*t* = 0.126	0.900^b^

The data are presented as the mean ± standard deviation.

MDD, major depressive disorder; HC, healthy controls; HAMD, Hamilton Rating Scale for Depression; HAMA, Hamilton Rating Scale for Anxiety; SSRIs, selective serotonin reuptake inhibitors; SNRIs, serotonin norepinephrine reuptake inhibitors; NaSSA, noradrenergic and specific serotonergic antidepressant; FD, frame-wise displacement.

^a^The P-value was obtained by Pearson Chi-square test.

^b^ The P-values were obtained by two-sample t-tests.

### MRI data acquisition

MRI data were collected on a 3.0-Tesla MR system (Discovery MR750, General Electric) with an 8-channel head coil. High-resolution three-dimension T1-weighted images were obtained sagittally with use of the following parameters: echo time (TE) = 3.2 ms; repetition time (TR) = 8.5 ms; flip angle (FA) = 12°; inversion time (TI) = 450 ms; matrix size = 256 × 256; field of view (FOV) = 256 mm × 256 mm; slice thickness = 1 mm, no gap; and 192 slices. Resting-state fMRI images were obtained axially with use of the following parameters: *TE* = 30 ms; *TR* = 2,000 ms; *FA* = 90°; matrix size = 64 × 64; FOV = 220 mm × 220 mm; slice thickness = 3 mm, slice gap = 1 mm; 35 interleaved slices; and 200 time points. Before the scanning, all subjects were instructed to keep their eyes closed, relax, move as little as possible, think of nothing in particular, and not fall asleep during the scans. During and after the scanning, we asked subjects whether they had fallen asleep to confirm that none of them had done so. All images were visually inspected to ensure that only images without visible artifacts (e.g., ghosting artifacts arising from subject movement and pulsating large arteries, metal artifacts, susceptibility artifacts, blooming artifacts) were included in subsequent analyses. All functional images were also checked to ensure that the whole cerebellum was covered.

### Functional magnetic resonance imaging data preprocessing

Statistical Parametric Mapping software (SPM12)^1^ and Data Processing & Analysis for Brain Imaging (DPABI)^2^ (51) were used to preprocess the resting-state fMRI data. The first 10 time points were deleted to enable the signal to reach equilibrium and the subjects to adapt to the scanning noise. The rest time points were then corrected for the acquisition time delay between slices. Next, realignment was done to correct the motion between time points. Head motion was indexed by translation in each direction and angular rotation on each axis. All subjects’ functional data were within the pre-defined thresholds (i.e., maximum translational or rotational motion < 2 mm or 2°). We also calculated frame-wise displacement (FD) estimating the volume-to-volume alterations in head position. There was no significant group difference in mean FD (*t* = 0.126, *P* = 0.900). Some nuisance variables (the linear drift, the Friston-24 head motion parameters, the spike time points with FD > 0.5, and the signals of white matter and cerebrospinal fluid) were regressed out from these data. The functional images were then band-pass filtered within a frequency range of 0.01–0.1 Hz. For the spatial normalization, structural images were initially co-registered to mean functional images; then the co-registered structural images were segmented and normalized to the Montreal Neurological Institute (MNI) space using the diffeomorphic anatomical registration through the exponentiated Lie algebra (DARTEL) technique (52). Finally, each functional image was normalized to the MNI space based on the deformation parameters derived during the above step and resliced into a 3-mm voxel.

### Functional connectivity calculation and feature extraction

Whole-brain functional connectivity analyses were conducted using GRETNA software^3^ (53). Network nodes were defined using the Shen brain atlas, which consists of 268 nodes and provides whole-brain coverage of the cortex, subcortex, and cerebellum (54). For each of the 268 nodes, the representative mean time course was obtained by averaging BOLD time courses over all voxels within the node. Then, we computed the node-by-node pairwise Pearson’s correlation coefficients and transformed them using Fisher’s z-transformation, resulting in a symmetric 268 × 268 correlation matrix for each subject. In the matrix, each element represents the strength of static functional connectivity between two individual nodes. To capture functional connectivity temporal dynamics, sliding time-window analysis was leveraged. Specifically, hamming windows (window size = 50, TR = 100 s, which satisfies the 1/f_0_ wavelength criterion for a minimum cutoff frequency of 0.01 Hz (55–58); window step = 1, TR = 2 s) were applied to each participant’s preprocessed functional data to obtain a set of BOLD signal windows (126 time windows for the current study). The above-mentioned whole-brain functional connectivity analysis was performed for each window, resulting in a total of 126 correlation matrices for each subject. Here, the standard deviations of the sliding-windowed correlation time series were used as a proxy of dynamic functional connectivity, where higher standard deviation represents greater signal dispersion from the average sliding-windowed correlation time series. For each subject, a 268 × 268 standard deviation matrix was constructed and each element in this matrix indexes the strength of dynamic functional connectivity between two nodes (59–61). For either of dynamic and static functional connectivity, a total of (268 × 267)/2 = 35,778 features were extracted and formed a high-dimensional feature representation.

### Functional connectivity-based classification

We utilized a linear kernel support vector machine (SVM), a method of supervised learning, as our classification algorithm as it allows the extraction of feature weights and shows resilience to over-fitting (62, 63). Firstly, feature selection was performed by using a two-tailed 2-sample *t*-test that assesses group difference in each functional connectivity feature. Only features with *P*-values smaller than a given threshold (0.01 for the main analysis) were retained, since these features differed significantly between the groups and were thus considered highly relevant to the class label. During the training step, the SVM identifies a decision boundary that separates the examples in the input space according to their class labels (i.e., patients vs. controls). Once the decision function is determined from the training set, it can be used to predict the class labels of new examples in the testing set. The linear kernel SVM has only one parameter *C* that controls the trade-off between allowing misclassifications and training error minimization. This parameter was fixed at *C* = 1 (a default value) for all cases.

A leave-one-out cross-validation (LOOCV) strategy was employed to estimate the performance of a classifier (64–66). Specifically, each of the subjects was in turn excluded, and the remaining subjects were included as the training set to train the classifier; the excluded subject was then used as the testing set to examine the capacity of the classifier to reliably differentiate between categories (i.e., patients vs. controls). This procedure was repeated for each subject to assess the overall accuracy, sensitivity and specificity, which could be utilized to quantify the performance of the classifier.


$$\text{Accuracy}=\frac{\text{TP}+\text{TN}}{\text{TP}+\text{FN}+\text{TN}+\text{FP}}$$

$$\text{Sensitivity}=\frac{\text{TP}}{\text{TP}+\text{FN}}$$

$$\text{Specificity}=\frac{\text{TN}}{\text{TN}+\text{FP}}$$

where TP (true positives) is the number of patients correctly classified; TN (true negatives) is the number of controls correctly classified; FP (false positives) is the number of controls classified as patients; FN (false negatives) is the number of patients classified as controls. In addition, the receiver operating characteristic (ROC) curve was plotted to illustrate sensitivity vs. 1-specificity across all possible values of discrimination threshold.

Statistical significance of the classification accuracy was determined by using permutation testing (67, 68). This testing examines the null hypothesis that the accuracy is obtained by change. In this analysis, the class labels (i.e., patients vs. controls) of the training set were permuted 5,000 times at random prior to training, and then the classification procedure was repeated to obtain 5,000 accuracy values. *P*-value was calculated as the proportion of accuracy values higher than the accuracy computed based on the true labels. The smaller the *P* is, the more reasonable we reject the null hypothesis that the accuracy is obtained by chance. Statistical significance level was determined at *P* < 0.05.

### Validation analyses

We carried out the following procedures to further assess the reproducibility of the results. First, global signal has been classically thought to reflect non-neuronal noise (e.g., movement, physiological, scanner-related) and GSR has been previously considered a standardized step during the preprocessing of resting-state fMRI data (69). However, global signal has recently also been found to represent neurobiologically relevant information (70–74). Thus, our main analysis was performed based on resting-state fMRI data without GSR. Given that GSR is still a highly debated topic (48), however, we also repeated our analysis using fMRI data with GSR. Second, we used a 2-sample *t*-test at a significance level of *P* < 0.01 to perform the feature selection. To estimate whether our main results were dependent on the choice of different thresholds, the classification procedure was repeated for connections selected by using two other significance levels (i.e., *P* < 0.5 and 0.001). Finally, given that different parcellation strategies may affect the results, we recalculated functional connectivity using two other parcellation schemes [i.e., AAL atlas with 116 nodes (75) and Random atlas with 1,024 nodes (76)] and reran the entire pipeline.

## Results

### Functional connectivity-based classifier performance

**FIGURE 1:**
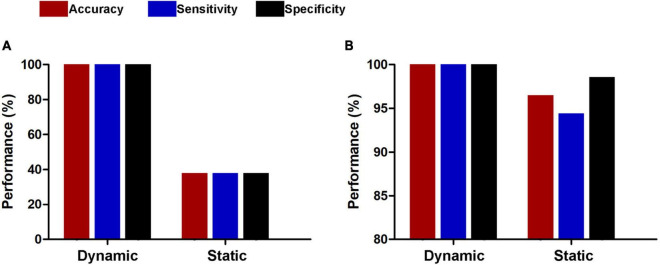
Performances of dynamic and static functional connectivity-based classifiers using resting-state fMRI data without **(A)** and with **(B)** global signal regression.

**FIGURE 2:**
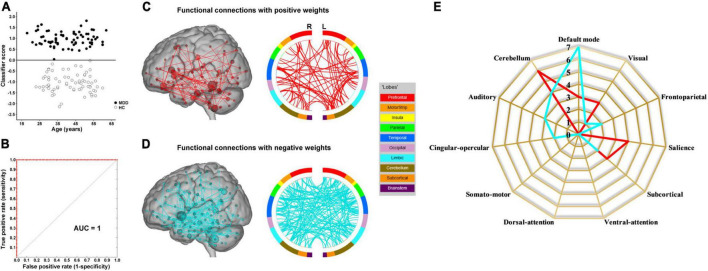
Dynamic functional connectivity-based classification. **(A)** Scatter plot showing classification scores of all the subjects. **(B)** Receiver operating characteristic (ROC) curve of the classifier. **(C)** High-degree nodes (degree ≥ 2, larger spheres indicate nodes with higher degree) and their connections in the positive network. **(D)** High-degree nodes (degree ≥ 2) and their connections in the negative network. **(E)** Fingerprints of the 25 highest-degree nodes summarized by overlap with canonical neural networks.

**FIGURE 3:**
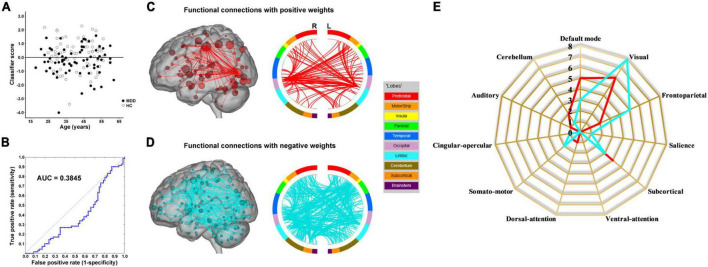
Static functional connectivity-based classification. **(A)** Scatter plot showing classification scores of all the subjects. **(B)** Receiver operating characteristic (ROC) curve of the classifier. **(C)** High-degree nodes (degree ≥ 50, larger spheres indicate nodes with higher degree) and their connections in the positive network. **(D)** High-degree nodes (degree ≥ 50) and their connections in the negative network. **(E)** Fingerprints of the 25 highest-degree nodes summarized by overlap with canonical neural networks.

The results of the linear kernel SVM classification between 71 patients and 71 controls based on dynamic and static functional connectivity are shown in Figure 1A. LOOCV of dynamic functional connectivity-based classification indicated that MDD patients could be discriminated from healthy controls with an excellent overall accuracy of 100% (a sensitivity of 100% and a specificity of 100%, permutation test with *P* < 0.0002) (Figure 2A). Figure 2B illustrates that the area under ROC curve (AUC) of the classifier was 1. However, the SVM classifier based on static functional connectivity yielded a poor overall accuracy of 38.0% (a sensitivity of 38.0% and a specificity of 38.0%, *P* = 0.9926) (Figure 3A) and the corresponding AUC was 0.3845 (Figure 3B).

**FIGURE 4:**
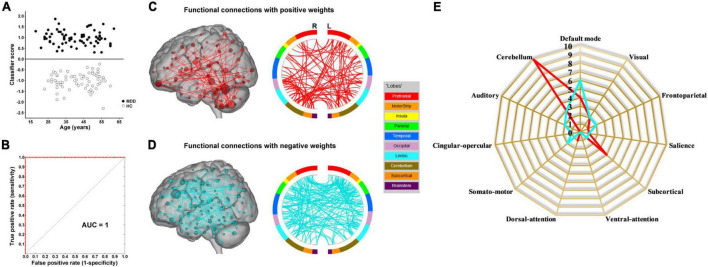
Dynamic functional connectivity-based classification using fMRI data with global signal regression. **(A)** Scatter plot showing classification scores of all the subjects. **(B)** Receiver operating characteristic (ROC) curve of the classifier. **(C)** High-degree nodes (degree ≥ 3, larger spheres indicate nodes with higher degree) and their connections in the positive network. **(D)** High-degree nodes (degree ≥ 3) and their connections in the negative network. **(E)** Fingerprints of the 25 highest-degree nodes summarized by overlap with canonical neural networks.

**FIGURE 5:**
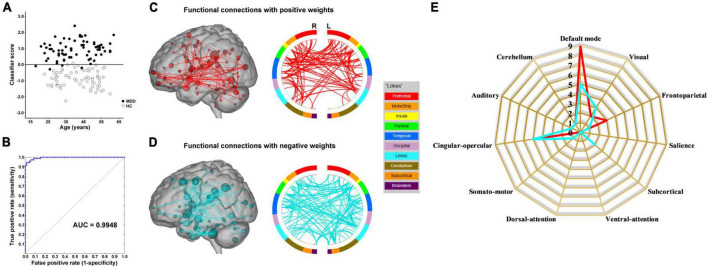
Static functional connectivity-based classification using fMRI data with global signal regression. **(A)** Scatter plot showing classification scores of all the subjects. **(B)** Receiver operating characteristic (ROC) curve of the classifier. **(C)** High-degree nodes (degree ≥ 8, larger spheres indicate nodes with higher degree) and their connections in the positive network. **(D)** High-degree nodes (degree ≥ 8) and their connections in the negative network. **(E)** Fingerprints of the 25 highest-degree nodes summarized by overlap with canonical neural networks.

The results of SVM classification using fMRI data with GSR are shown in Figure 1B. Both dynamic and static functional connectivity-based classifiers achieved good performances in the discrimination of patients and controls, with the former better than the latter. For dynamic functional connectivity, the overall accuracy was 100% (a sensitivity of 100% and a specificity of 100%, *P* < 0.0002) (Figure 4A) and the corresponding AUC was 1 (Figure 4B). For static functional connectivity, the overall accuracy was 96.5% (a sensitivity of 94.4% and a specificity of 98.6%, *P* < 0.0002) (Figure 5A) and the AUC was 0.9948 (Figure 5B).

**FIGURE 6:**
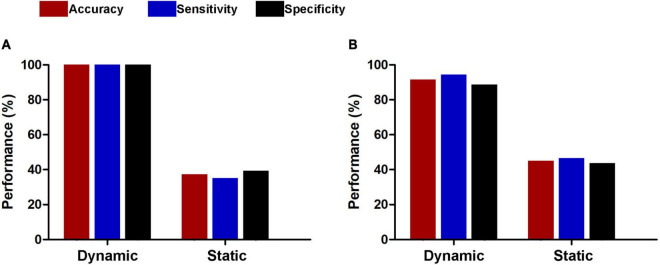
Classification performances of dynamic and static functional connectivity-based classifiers using subsets of connections selected by thresholds of *P* < 0.05 **(A)** and 0.001 **(B)**.

**FIGURE 7:**
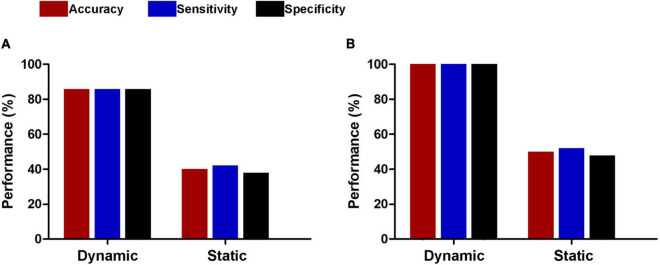
Classification performances of dynamic and static functional connectivity-based classifiers using AAL atlas with 116 nodes **(A)** and Random atlas with 1,024 nodes **(B)**.

For subsets of connections selected by thresholds of *P* < 0.5 and 0.001, the classification results were similar to those at the threshold of *P* < 0.01, i.e., dynamic functional connectivity classifiers (threshold of *P* < 0.5: accuracy = 100%, sensitivity = 100%, specificity = 100%, *P* < 0.0002, and AUC = 1; threshold of *P* < 0.001: accuracy = 91.5%, sensitivity = 94.4%, specificity = 88.7%, *P* < 0.0002, and AUC = 0.9820) performed much better than static functional connectivity classifiers (threshold of *P* < 0.5: accuracy = 37.3%, sensitivity = 35.2%, specificity = 39.4%, *P* = 0.9992, and AUC = 0.3499; threshold of *P* < 0.001: accuracy = 45.1%, sensitivity = 46.5%, specificity = 43.7%, *P* = 0.6784, and AUC = 0.4773) (Figure 6). In addition, we found that our main results were reproducible after considering the effects of different parcellation strategies (Figure 7). For AAL atlas with 116 nodes, although the overall accuracy of dynamic functional connectivity classifier reduced to 85.9% (sensitivity = 85.9%, specificity = 85.9%, *P* < 0.0002, and AUC = 0.9375), it was higher than that of static functional connectivity classifier (accuracy = 40.1%, sensitivity = 42.3%, specificity = 38.0%, *P* = 0.9952, and AUC = 0.3718). For Random atlas with 1,024 nodes, the dynamic functional connectivity classifier (accuracy = 100%, sensitivity = 100%, specificity = 100%, *P* < 0.0002, and AUC = 1) exhibited a better classification power than the static functional connectivity classifier (accuracy = 50%, sensitivity = 52.1%, specificity = 47.9%, *P* = 0.8042, and AUC = 0.4575).

### Classification network anatomy

The weight vector provided relevant information about the contribution of functional connections to classification. Specifically, functional connections with positive weights contributed to the identification of MDD patients, whereas connections with negative weights contributed to the identification of healthy controls. The dynamic functional connectivity-based classification identified positive and negative networks consisting of 95 connections with positive weights and 170 connections with negative weights, respectively (Figures 2C,D). Network anatomies for both networks were complex and included connections between nodes across the brain. Of the 25 nodes showing the highest degree (i.e., number of functional connections) in the positive network, 6 were within the cerebellum, 4 the salience network, 3 the default mode network, 3 the visual network, and 3 the subcortical network (Figure 2E). Of the 25 highest-degree nodes in the negative network, 7 were within the default mode network, 4 the cerebellum, and 3 the auditory network (Figure 2E). The static functional connectivity-based classification identified positive and negative networks consisting of 1,719 and 2,048 connections, respectively (Figures 3C,D). Of the 25 highest-degree nodes in the positive network, 6 were within the visual network, 5 the default mode network, and 4 the subcortical network (Figure 3E). Of the 25 highest-degree nodes in the negative network, 8 were within the visual network, 5 were within the frontoparietal network, 3 the default mode network, and 3 the subcortical network (Figure 3E).

Network anatomies of the classification using fMRI data with GSR are demonstrated in Figures 4C–E and Figures 5C–E. The dynamic functional connectivity-based classification identified positive and negative networks consisting of 137 and 186 connections, respectively (Figures 4C,D). Of the 25 highest-degree nodes in the positive network, 10 were within the cerebellum, 4 the default mode network, and 4 the subcortical network (Figure 4E). Of the 25 highest-degree nodes in the negative network, 6 were within the default mode network and 3 the cerebellum (Figure 4E). The static functional connectivity-based classification identified positive and negative networks consisting of 390 and 376 connections, respectively (Figures 5C,D). Of the 25 highest-degree nodes in the positive network, 9 were within the default mode network, 5 the cingulo-opercular network, and 3 the frontoparietal network (Figure 5E). Of the 25 highest-degree nodes in the negative network, 5 were within the default mode network, 5 the cingulo-opercular network, and 3 the visual network (Figure 5E).

## Discussion

Based on machine learning approach, the present study demonstrated that MDD patients can be distinguished from healthy controls with excellent and stable classification accuracy using whole-brain dynamic functional connectivity irrespective of whether or not GSR was performed. Brain regions with dynamic connectivity contributing the most to the identification of patients were mainly and reliably located within the default mode and subcortical networks as well as the cerebellum, and regions with connectivity contributing to the identification of controls were predominately located within the default mode network and the cerebellum. In contrast, the classifiers based on static functional connectivity exhibited unstable classification performances, i.e., a low accuracy without GSR while a high accuracy with GSR; moreover, there was a considerable variability in the distribution of brain regions with static connectivity most informative for classification.

Traditional resting-state functional connectivity is assumed to have static nature and thus reflects mean correlation patterns of spontaneous BOLD signal fluctuations between regions within a typical resting-state fMRI experiment, which cannot depict the full extent of intrinsic neural activity given that human brain is expected to integrate, coordinate and respond to internal and external stimuli dynamically across time. On top of this, resting-state functional connectivity has recently been proved to have dynamic behavior (17, 22), resulting in a considerable amount of studies directed to assessing and characterizing its dynamics (18–20). Among several dynamic functional connectivity methods, the variance measure which we used in this study reflects the degree of the meaningful functional connectivity variations, and it has been demonstrated to contain the most useful information that can be applied to detect inter-individual differences (59). High variance value indicates that the functional connectivity strength between regions greatly fluctuates in and out of synchrony, reflecting more flexible brain communication between these regions. Collectively, static and dynamic functional connectivity analyses capture different aspects of inter-region functional communication and might provide complementary information. Using fMRI data with GSR, the classification accuracy based on static functional connectivity is comparable to that reported in a prior study (96.5 vs. 94.3%) (77), while the accuracy reduced dramatically without GSR, suggesting a low robustness of the static functional connectivity-based classifier. However, the current observation of excellent and stable performance of dynamic functional connectivity-based classification of depression adds important context to the growing literature on the relevance of dynamic functional connectivity.

The brain’s default mode network has been linked to emotional processing, self-referential mental activity, episodic memory retrieval, and internal-directed attention (78). Dysfunction of the default mode network has been widely documented in depression (79, 80), along with its association with core clinical manifestations of this disorder such as depressive rumination (81). With respect to functional connectivity changes of the default mode network in MDD patients, most previous studies have reported hyperconnectivity (11, 81–84), while some studies have found hypoconnectivity (85) as well as both increase and decrease (86, 87). These inconsistencies may arise from limited statistical power, flexibility in data analysis (88), and heterogeneity in clinical characteristics. In this study, we found that brain regions within the default mode network had dynamic functional connectivity that was most informative in distinguishing the samples. These findings, in conjunction with those of previous studies, raise the possibility that functional dysconnectivity of the default mode network may be a trait of depression, highlighting a prominent role of this circuitry in the pathophysiology of MDD.

There is consistent evidence that the cerebellum is critically involved in multiple high-order functions including cognition and emotion that exist above and beyond low-order motor function (89, 90). More recently, the clinical relevance of the cerebellar damage in depression has attracted intense interest from researchers (91), given the extant findings of structural and functional impairments of the cerebellum in patients with MDD (92–95). Neuroimaging studies have established that the cerebellum is a multifactorial structure that can be divided into functionally separate and topographically organized subsystems (96, 97), connections of which form cerebellar-cerebral circuits mediating executive control, default mode, affective, and motor functions (90, 98, 99). Previous studies using resting-state fMRI have produced mixed findings that MDD patients show both decreased and increased functional connectivity between cerebellum and widespread cerebral regions relative to healthy controls (100–103). Moreover, Ma et al. have found that a high classification accuracy of 90.6% was achieved by selecting altered cerebellar-cerebral functional connectivity as features to discriminate MDD patients from healthy subjects (104), which is compatible with our observation that abnormal dynamic functional connectivity of the cerebellum was most informative in distinguishing MDD and control subjects. Combined, these findings suggest that disruption of cerebellar-cerebral functional interactions may serve as one of the depression-specific neurobiological characteristics.

The subcortical network consists of striatum, thalamus, hippocampus, and amygdala, which are implicated in a variety of functions such as emotional regulation, reward processing, motivational management, cognitive processes, movement regulation, and memory recall. Many studies have revealed structural and functional alterations in these subcortical regions in depressed patients (80, 92, 105–110), which may lead to deficits of the relevant functions in MDD. Moreover, complex subcortical connections constitute multiple cortical-subcortical circuits including frontal-striatal, prefrontal-hippocampal, prefrontal-amygdala, and frontal-thalamic circuits (111–113). Structural and functional connectivity impairments of these cortical-subcortical circuits are considered to be potential neuropathological targets in MDD (35, 114–117). Our findings of subcortical dynamic functional connectivity exhibiting high discriminative power in classification complement and extend previous literature on the role of subcortical network in understanding the neural mechanisms of depression.

Some limitations of this study should be acknowledged. First, our results may be contaminated by the confounding factors such as medication use and/or long illness duration. Future studies using a sample of drug-naive first-episode patients with MDD are warranted to confirm the reliability of our findings. Second, during the resting-state fMRI scans, subjects’ levels of drowsiness or vigilance have been shown to influence dynamic functional connectivity (118). Here, we did not measure these variables and thus cannot rule out their potential effects. However, the variance measure that we utilized as the summary index of dynamic functional connectivity has been demonstrated to exhibit good test-retest reliability (59), which may partly alleviate the concerns of noise interference. Third, it should be noted that patients with anxiety disorders were excluded. Since anxiety is frequently comorbid with MDD, this reduces the generalizability of the findings to the general population with MDD. Fourth, the lack of data from an independent sample precludes us from performing an external validation analysis. Fifth, artifacts from cardiac and respiratory noise are prevalent in resting-state fMRI analyses. Thus, an advisable pre-processing step is to remove physiological noise from the data using simultaneously collected pulse and respiration data. However, physiological data were not collected in this study. Sixth, MDD patients had a trend toward a lower educational level than healthy controls, which may influence our interpretation. Seventh, the resting-state fMRI scan duration is relatively short and may lead to less stable results. Thus, future studies are needed to validate the results by increasing the fMRI scan length. Finally, we only focused on the discrimination between MDD patients and healthy controls, but it is unclear whether the application of SVM to whole-brain dynamic functional connectivity would differentiate MDD patients from patients with other mental disorders. Further investigations could address this issue by including a third group of individuals with a mental disorder other than MDD.

In conclusion, this study successfully demonstrates the feasibility of machine learning approach toward the objective and accurate diagnosis of MDD patients by using whole-brain dynamic functional connectivity as input features. The classification accuracy and stability of dynamic connectivity-based classifier were superior to those of static connectivity-based classifier. Brain regions with the most discriminating dynamic connectivity were mainly and reliably located within the default mode network, cerebellum, and subcortical network. We believe that the current findings will be helpful for a better understanding of the neural basis of MDD as well as for the development of effective computer-aided diagnosis tools in clinical settings.

## Data availability statement

The raw data supporting the conclusions of this article will be made available by the authors, without undue reservation.

## Ethics statement

The studies involving human participants were reviewed and approved by the Ethics Committee of Shanxi Cardiovascular Hospital. The patients/participants provided their written informed consent to participate in this study.

## Author contributions

HN, WL, and TC designed the research, analyzed the data, and wrote the manuscript. GW, QH, RH, TL, and FZ conducted the clinical evaluation and acquired the clinical and MRI data. TC reviewed the manuscript for intellectual content. All authors contributed to and have approved the final manuscript.

## Footnotes

1
http://www.fil.ion.ucl.ac.uk/spm

2
http://rfmri.org/dpabi

3
http://www.nitrc.org/projects/gretna
